# Blind, Deaf, Defective, and Epileptic Children: Their Education and Training

**Published:** 1917-07-21

**Authors:** 


### BLIND, DEAF, DEFECTIVE, AND EPILEPTIC CHILDREN.

### Their Education and Training.

THE essence of the Elementary Education Acts (1870 to 1914), as applied to blind, deaf, defective, and epileptic children, is that parents of such children must cause them to receive elementary education whatever the distance to the nearest special school, and notwithstanding that they may be called upon to contribute towards the expenses incurred by the school authorities. The Acts give local authorities full power to establish special schools, and to subsidise schools managed by charitable bodies. In point of fact, fees are not now required of parents, and local authorities have efficiently responded to the call upon them to provide suitable opportunities for the education of abnormal children.

The Acts of Parliament dealing with this important matter, as is the way of Acts of Parliament, are drafted in language which sounds cold and almost unsympathetic. That part of the Acts which is translated into the recently issued regulations of the Board of Education has been imbued with a spirit of kindliness that is especially suited to a subject dealing with the children of the nation who at the outset of life have to bear a heavy handicap.

The Board of Education in a prefatory memorandum praises what has been done, and in encouraging further progress looks far ahead. In this memorandum it is recognised that during the war it would be inopportune to introduce into the regulations any new requirements; the revision is therefore confined to the incorporation of minutes passed by the Board since the last issue of the regulations (in 1909), and to such changes as appear desirable to bring the regulations more into accord with the best existing practice. It is thought that most existing schools comply with the new regulations, and in any case full allowance will be made where they do not entirely do so, and modifications will not be required during the continuance of the war.

The minutes referred to deal with the status of persons recognised as head-teachers, to increases in capitation grants paid to schools, and to expenditure of local authorities in respect of boarding mentally defective children. Thus during the last seven years the regulations have remained substantially unaltered. But in the same period the schools have not been unprogressive. On the contrary, the Board of Education pays a handsome tribute, and places on record the fact that a remarkable development has taken place in the character, scope, and number of the special schools provided for abnormal children.

New types of school have come into existence, and the number of children in attendance has increased by 50 per cent. To the development of an effective School Medical Service is attributed this remarkable growth, the facilities for medical diagnosis thus rendered available have led not only to the collection of more complete information as to the children for whom special provision is required, but also to more effective consideration of the needs of such children, and the nature of the provision necessary for their education and welfare. Moreover, the significance of the term "physically defective" has been more clearly appreciated, and now includes, in addition to the permanently disabled, tuberculous or delicate children who cannot attend public elementary schools without injury, but for whom attendance at a special open-air school may mean not only instruction suited to their particular needs, but also restoration to health. Thus the mission of the special schools has been extended from making the best of a bad thing to preventing, on a much greater scale, a worse thing, and may claim in character to be preventative, curative, and educative, instead of merely curative and educative. The life of the young is so precious to the nation at the present time that this economical feature of the work of caring for defective children cannot be too gratefully recognised.

The open-air school has been constantly encouraged by the Board of Education, and it must not be supposed that the beneficial conditions provided in this way are used exclusively for children suffering from tuberculosis, debility, or pre-tubercular conditions, for the benefits of instruction under open-air conditions have not been confined to delicate children, but are being increasingly extended to all types of special school. Moreover, in the instruction the importance of manual work is increasingly emphasised, and instead of being regarded as a distinct subject it is tending to become increasingly the basis upon which the whole work of the school is built up.

One of the chief alterations or elaborations in the regulations shows us that an important part of the work of the School Medical Service relates to the welfare of the child after admission to the special school. It is provided that blind and deaf children shall be examined from time to time by a medical practitioner of special experience, and that a constant watch over the general physical condition of all children, not merely those requiring medical attention, shall be part of the duties of the service.

On the educational side, considerable attention has recently been given to the problem of the training of teachers for schools for mentally defective children, and a special article in the regulations deals with the curriculum for defective and epileptic children in which the need for training the residual senses of the blind is particularly emphasised.

The building rules for schools have been added to considerably since the last issue of the regulations. Very wisely it is required that schools for various types of afflicted children shall not be held in the same building unless structurally separated for each type.

The schools certified under the Elementary Education Acts provide only for children in special schools up to the age of sixteen; it is becoming increasingly recognised that instruction and training should be continued beyond that age if the scholars are to derive full benefit from their school attendance, and to be afforded a reasonable opportunity of earning a livelihood. The Board therefore encourage, by means of a grant, institutions undertaking this post-school instruction, and have prepared regulations detailing conditions required before pecuniary assistance will be given. As it is anticipated that the majority of the institutions recognised under these regulations will be under the same management as the special schools, it has been provided, to secure simplicity of administration, that many of the provisions of the school regulations will also apply to the training institutions.

